# Bilateral knee septic arthritis: Was it from sternal wound?

**DOI:** 10.1002/ccr3.1727

**Published:** 2018-07-17

**Authors:** Han Hong Chong

**Affiliations:** ^1^ Trauma & Orthopaedic Lincoln County Hospital United Lincolnshire Hospitals NHS Trust Lincoln UK

**Keywords:** bilateral, infection, orthopedic, septic arthritis, wound infection

## Abstract

Polyarticular septic arthritis is a rare but life‐threatening condition and should not be underestimated. Clinicians should look for potential source of infection, either local spread or hematogenous distribution. However, this should not delay the main treatment for septic arthritis.

**Learning Points**

1. Although rare, acute‐onset polyarticular joint swelling and pain should be considered as possible septic arthritis alongside relevant clinical history.
2. Potential source of infection should be investigated including distant wound infection; however, it should not delay the treatment of septic arthritis.
3. Surgical washout remains mainstay of treatment to reduce organism load in native joint and to enhance the efficacy of antibiotics used.

## 1 INTRODUCTION

An acutely painful swollen joint is a common medical presentation with a broad differential diagnosis for consideration. The less common but serious complication being septic arthritis (SA) which one must consider. The incidence of SA in Western Europe is 4‐10 per 100 000 patients per year with approximately 50% cases involving the knee joint.^1^ Polyarticular SA is uncommon with an estimated 15% of all SA cases reported. A high mortality rate of 50% in polyarticular SA highlights the importance of early recognition and intervention.^1^

This case also highlights an unusual presentation of bilateral SA. The aim of this report was to raise the awareness and hence importance of early recognition of SA, its possible link to distant wound infection.

## 2 CASE PRESENTATION

A 65‐year‐old gentleman presented to the emergency department (ED) at night with a history of acute‐onset atraumatic bilateral knee pain and swelling. His past medical history includes bicuspid aortic valve replacement and subsequent revision of his aortic valve replacement 4 weeks prior to the onset of knee pains. He was diagnosed with sternal surgical wound infection (clinically erythematous skin with possible discharge) 4 days prior to hospital attendance with associated left knee pain and swelling. A diagnosis of wound infection with reactive arthritis was made by his general practitioner (GP), and the patient was commenced on a course of clarithromycin due to penicillin allergy. The wound infection appeared to be responsive to oral antibiotic treatment. However, his left knee symptoms gradually worsened and greatly affected his mobility; 12 hours before ED attendance, he developed acute right knee pain and swelling with associated generalized fatigue. Clinical observation raised concerns of sepsis with a pyrexia of 39°C and tachycardia of 100 beats per minute. Both of his knees were diffusely swollen, warm, and extremely tender to palpate. He did not tolerate any range of movement of his knees. Hip and ankle joints were normal, and there was no appearance of cellulitis. He was also reviewed by the medical team, and other common sources of sepsis including chest or urine infections were ruled out.

### 2.1 Investigation

**Figure 1:**
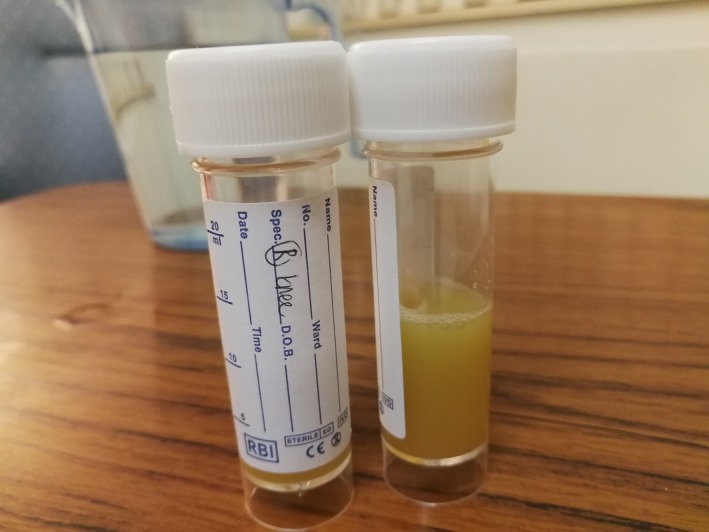
Right knee synovial fluid from needle aspiration

**Figure 2:**
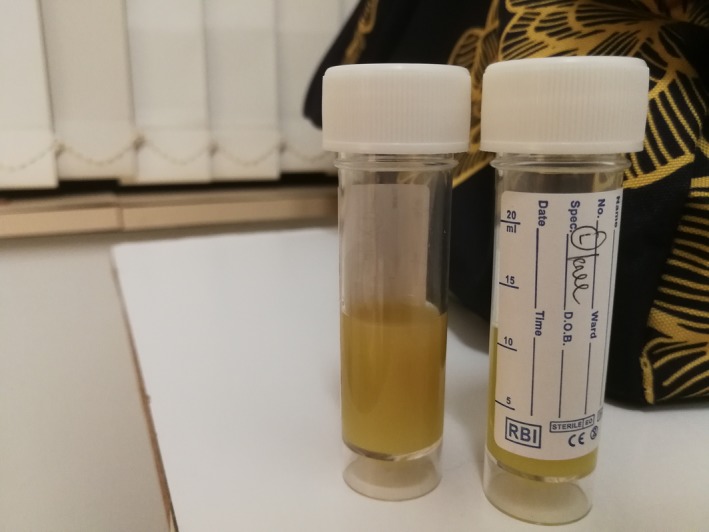
Left knee synovial fluid from needle aspiration

On admission, his blood test showed raised inflammatory markers, with white blood cells of 15.9 × 109 L and C‐reactive protein (CRP) of 288 mg/L. His knee X‐rays revealed no bony pathology otherwise. Aspiration of both knees was performed under sterile technique on the ward prior to commencing antibiotics—cloudy thick pus was drained from both knees (Figures 1 and 2). Urgent microscopy and gram stain did not show any organisms.

### 2.2 Differential diagnosis

The patient's initial presentation to his general practice with acute unilateral atraumatic knee pain and swelling raised suspicion of reactive arthritis secondary to a wound infection. His subsequent presentation to the emergency department with bilateral knee symptoms and inability to mobilize associated with systemic symptoms including pyrexia raised the clinical suspicion of bilateral SA. Raised white blood cell and C‐reactive protein suggest SA as the most likely diagnosis, although both knee aspirations were negative.

### 2.3 Treatment

**Figure 3:**
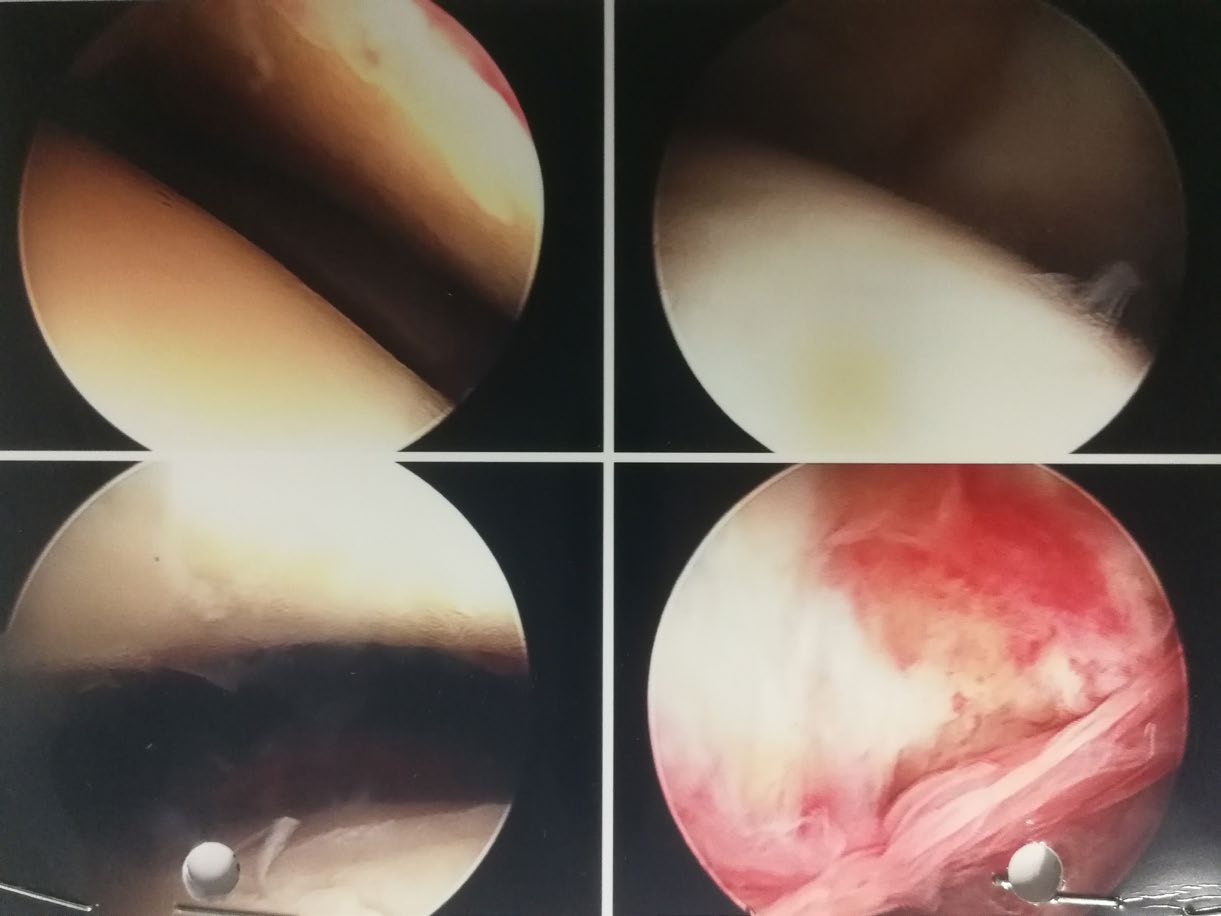
Intra‐operative arthroscopic photos. Bottom right shown active synovitis

This patient was started on combination of antibiotics treatment according to the local hospital guidelines. He then proceeded to have bilateral knee arthroscopies and washout which revealed more pus collections and active synovitis (Figure 3). Synovial fluid samples were obtained and sent for culture. He was treated with a total 3‐week course of intravenous antibiotics and underwent a total of four knee joint washouts. A total of six synovial fluids and two intra‐articular soft tissues were sent for culture—none of which grew any active microorganisms. The possibility of infective endocarditis was also considered in view of the recent revision of his prosthetic valve. He had a total of four blood cultures collected at separate times, all of which were negative. Inpatient echocardiogram and subsequent transesophageal echocardiogram (TOE) showed no vegetation on his prosthetic heart valve.

### 2.4 Outcome and follow‐up

After repeated washouts and a prolonged course of IV antibiotics, the patient demonstrated full clinical recovery also evident with improvements in his inflammatory markers. He was then discharged with a further 2‐week course of oral antibiotics along with an outpatient clinic follow‐up. He underwent subsequent reviews by a cardiothoracic surgeon and cardiologist in view of his recent prosthetic heart valve revision surgery which was satisfactory.

## 3 DISCUSSION

Polyarticular joint involvement of SA is an uncommon joint condition. The most common organisms associated with SA are Staphylococcus Aureus, followed by the Streptococci group.^1^, ^2^, ^3^, ^4^

Septic arthritis can occur in any age group, with the extremities of both age groups being more susceptible to it. History of joint diseases such as rheumatoid arthritis, osteoarthritis, systemic lupus erythematosus, and trauma are known to be predisposing factors for developing SA. Other systemic causes that increase the risk of SA include immunosuppressive diseases (diabetes, malignancy, renal, or liver disease), endocarditis, skin infection, and intravenous drug use.^1^, ^2^, ^3^, ^4^ In this case, our patient had a recent revision of his aortic valve replacement (noninfective) and postoperative sternal wound infection after four weeks. He was otherwise immunocompetent with no previous joint disease. There have been cases reported in the literature on the association between infective endocarditis and polyarticular septic arthritis. However, to date, there has been no literature on bilateral knee septic arthritis with either distance wound infection or prosthetic heart valve.

The gold standard for investigating SA is synovial fluid aspiration along with blood tests and cultures.^1^ The sample should be sent for urgent Gram stain and culture for antibiotic sensitivity. There are multiple literatures published on the choice of synovial fluid test. Unfortunately, the sensitivity for Gram stain ranges from 29% to 50% and for culture which is only as sensitive as 76%.^5^ In our case, a total of five synovial fluid samples were obtained during the initial needle aspiration on admission and a further four from knee washouts intra‐operatively; none of the samples demonstrated growth of any organisms. SA should be suspected and treated accordingly if important clinic features such as joint pain, swelling, reduction in the range of movement, fever, and raised inflammatory markers are present despite a negative synovial fluid result.

The hypothetical cause of SA in this case report according to the sequence of events is highly suggestive of a sternal wound infection as the source of infection. The patient had begun developing left knee SA symptoms 4 days following the diagnosis of a sternal wound infection which, due to a delayed presentation, might have resulted in a hematogenous spread to his right knee. The initial course of oral antibiotics used to treat his sternal wound infection may have contributed toward the negative cultures from his bilateral knee needle aspirations. Unfortunately, this case report is limited by negative culture result from initial sternal wound swab, multiple synovial fluid samples, soft tissue samples, and blood cultures.

Native joint washouts and intravenous antibiotics remain the main treatment plan for native knee SA. There are no established trials on the most effective surgical management of SA—aspiration, arthroscopic or open washout.^6^ The main objective of surgical intervention is to reduce organism load in the native joint to enhance the efficacy of antibiotics used which was proven to be clinically effective in this case.

## CONFLICT OF INTEREST

None declared.

## AUTHORSHIP

HHC: had gain consent from the patient and wrote up the full case report.
